# Metal-on-Metal Hips: Ten-Year Clinical and Radiographic Outcomes of the ADEPT Metal-on-Metal Hip Resurfacing and Modular Total Hip Arthroplasty

**DOI:** 10.3390/jcm12030889

**Published:** 2023-01-22

**Authors:** Fabio Mancino, Michael A. Finsterwald, Christopher W. Jones, Gareth H. Prosser, Piers J. Yates

**Affiliations:** 1 Department of Orthopaedics, The Orthopaedic Research Foundation of Western Australia (ORFWA), Fiona Stanley Hospital, 6150 Perth, Australia; 2 Curtin Medical School, Faculty of Health Sciences, Curtin University, 6102 Perth, Australia; 3 UWA Medical School, University of Western Australia, 6009 Perth, Australia

**Keywords:** metal-on-metal, hip resurfacing, total hip arthroplasty, metal ions, trunnionosis, large diameter head, hip dislocation

## Abstract

Background: The aim of this study is to update the 10-year follow-up survivorship and metal ions levels of a cohort of metal-on-metal (MoM) hip resurfacing (HR) and large-diameter-head (LDH) total hip arthroplasty (THA). Methods: The study is a retrospective analysis of prospectively collected data that compared the outcomes of 24 MoM HR (21 patients) and 15 (11 patients) modular LHD MoM THA at >10 years follow-up. Baseline characteristics as well as intraoperative and postoperative information were collected, including complications, revisions, clinical and radiographic outcomes, and serum metal ions level (Cobalt, Chromium). Metal ion levels were compared using a two-tailed unpaired t-test and Wilcoxon signed-rank test (jamovi v2.3.3.0, Sydney, NSW, AU). Results: No significant differences were detected in gender, BMI, and ASA score between the two groups. Patients in the modular THA group were significantly older (57 years vs. 46 years; *p* < 0.05). The HR overall survivorship was 91.7% (22 of 24 hips) with survivorship from implant failure and/or aseptic loosening and/or metal debris related 100% of problems. The modular THA overall survivorship was 86.7% (13 of 15 hips) with survivorship from implant aseptic loosening and metal ions complications of 93.4% (14 of 15 hips). No significant difference was noted when comparing clinical outcomes. Metal ions were significantly lower in the HR group (Co 25.8 nmol/L vs. 89 nmol/L; *p* < 0.001–Cr 33.5 nmol/L vs. 55.2 nmol/L; *p* = 0.026). Conclusion: Both implants reported excellent and comparable clinical outcomes at >10 years follow-up. The Adept HR reported remarkable survivorship, in line with the registry data, proving once again its reliability in young active males. The modular LDH THA, despite being discontinued, presented higher reliability and a lower failure rate when compared with similar withdrawn MoM implants. Trunnionosis did not appear to be a significant problem in this particular modular design.

## 1. Introduction

Total hip arthroplasty (THA) is one of the most successful surgical procedures of the past 50 years. It is performed worldwide with excellent results, and it has recently been proclaimed “the operation of the century” [1]. Large diameter femoral head (LDH) metal-on-metal (MoM) THA and MoM hip resurfacing (HR) were introduced with high hopes for their tribological properties, reducing polyethylene-wear related osteolysis while providing increased range of motion (ROM) and joint stability [2,3].

HR was introduced in the 1970s as a bone-preserving alternative to THA for younger, more active patients with osteoarthritis [3,4]. After the failure of the metal-on-polyethylene HR bearing for high volumetric wear, polyethylene debris, osteolysis, and loosening [3,4,5], MoM bearings were developed to allow larger-diameter femoral resurfacing with thinner acetabular components. Since then, significant benefits have been associated with HR compared to THA, including lower dislocation rate, improved restoration of normal hip biomechanics, reduced leg length errors (LLD), preservation of femoral bone stock, and easier revision surgery [6,7]. At its peak in 2006, HR was used in 10.4% of hip arthroplasty cases [8]. HR results are excellent in carefully selected patients treated by experienced surgeons using the few well performing implants, especially in very highly active patients [9,10], with a recently reported survivorship of 88.9% at 22 years follow-up (11,382 MoM HR; ≤50 years age) [10].

However, problems were reported, including sensitivity to metal ions, progressive thinning of the femoral neck, femoral neck fracture, and avascular necrosis (AVN) of the femoral head [11,12,13]. In addition, some HR and LDH THA led to unexpected failure rates of up to 30% at seven years [14,15,16], with increased cases of adverse reaction to metal debris (ARMD) and aseptic lymphocyte-dominated vasculitis-associated lesion (ALVAL), osteolysis, trunnionosis, increased blood metal ions, and metallosis [17]. These implants fell rapidly out of favor and a few designs were withdrawn from the market [5,18,19]. This was especially true for the LDH MoM implants, where the additional trunnion junction articulating with a LDH appeared to be a significant cause of failure due to corrosion and metal ions release.

The short-term clinical and radiographic results of a comparative series of Adept (Finsbury Orthopaedics; subsequently MatOrtho, UK; 2005 onwards) LDH MoM modular THA and HR has been previously reported with promising findings [20]. The aim of this study was to update the results at >10 years reporting on our Adept LDH MoM hip arthroplasty failure rates, clinical outcomes, radiographic findings, and metal ion levels. The hypothesis was twofold, namely (1) that HR was associated with higher survivorship, higher functional outcomes, and lower metal ions levels compared to the modular LDH THA, (2) and that the modular THA, despite being recalled, was associated with better survivorship compared with similar withdrawn implants.

## 2. Materials and Methods

This is a retrospective study, using longitudinally maintained data, of 43 patients (50 hips) that underwent HR or THA using Adept implant between 2007 and 2011 by two experienced, fellowship-trained arthroplasty surgeons (GP, PY). The specifics of each implant were recorded at time of index surgery, along with baseline demographics characteristics of the patients (body mass index (BMI), age, gender, reason for surgery). Ethical approval for the retrospective review was granted from the Institutional Human Research Ethics Committee and informed consent was obtained.

This study included 24 MoM HR (21 patients) and 15 (11 patients) modular LHD MoM THA for which the results at a mean of 28 months follow-up had been previously reported [20]. Two patients are included for survivorship in both groups as they were originally treated with HR for primary OA, experienced a periprosthetic fracture within 6 months postoperatively, and were revised with LHD MoM THA. At the 10-year follow-up, in the resurfacing group, 2 patients (2 hips) had died, and 2 patients (2 hips) could not attend the clinical appointment, leaving 24 MoM hip resurfacing (21 patients) for final analysis (86%). In the modular THA group, 4 patients (6 hips) had died, and 3 patients (3 hips) could not attend follow-up for a minimum 10 years, leaving 15 modular MoM THA (63%). Among the patients who were not available for 10-years follow-up (5 patients/5 hips), the implants were well fixed at the last clinic visit and when contacted by telephone patients reported that they did not undergo further revision surgeries. Therefore, these hips were included in the survivorship analysis, leaving 93% of the HR initial cohort (26 of 28 hips) and 75% of the initial modular MoM THA cohort (18 of 24 hips). Complete clinical and radiographic data were available at minimum 10-year follow-up (mean, 11.7 years; range, 10–14.2 years) for 22 HR (21 patients) and 13 MoM THA (9 patients). Further details on baseline information are addressed in Table 1.

The methods with respect to surgical techniques, patient follow-up, clinical hip scores, radiolucent lines, implant fixation, and radiographic analyses were similar to our earlier study [20]. The surgical technique was as described by McMinn et al. [3,4]. When a modular implant was used, the femoral component was a Corail stem (DePuy, Warsaw, IN, USA), a tapered stem made of forged titanium, aluminum, and vanadium alloy (TiAl6V4) extensively coated with hydroxyapatite combined with the modular CoCr Adept femoral head. The modular system is characterized by increasing head size and concomitant larger acetabular component size, plus the option of a high offset neck. HR femoral components were cemented using medium-viscosity Simplex™ P cement (Stryker, Kalamazoo, US).

The main indication for surgery was primary hip osteoarthritis in both groups (HR 79.2%, THA 66.7%). Further information on indication of index surgery is addressed in Table 2.

Each patient received intravenous perioperative antibiotic prophylaxis, multimodal pain management, and routine prophylaxis for thromboembolic disease with intraoperative calf compressors, early mobilization, and aspirin (150 mg/d for 6 weeks). All patients were permitted weightbearing as tolerated using crutches or a cane as necessary starting from day of surgery, or postoperative day 1 (POD 1) when surgery was performed in the afternoon. The rehabilitation protocol with active ROM exercises and progressive weightbearing was started in the surgical ward.

Patients were followed clinically and radiographically at the preoperative visit, at 2 weeks after surgery for the wound check, then at 6 weeks, 6 months, 12 months, and yearly thereafter. Hip function was assessed using the well-established Oxford Hip Score (OHS) [21], University of California and Los Angeles Activity Score (UCLA) [22], and Western Ontario and McMaster Universities Osteoarthritis Index (WOMAC) [23]. Radiographic review was performed by an independent arthroplasty fellowship-trained orthopaedic surgeon. A plain antero-posterior (AP) radiograph of the pelvis, in addition to AP and lateral of the involved hip, were performed prior to discharge and on a yearly basis to evaluate and assess radiolucent lines, osteolysis, signs of fracture, component loosening, cup angle, femoral offset, implant migration, neck thinning, cortical hypertrophy, stress shielding, femoral component subsidence, and implant osseointegration according to the criteria of Engh et al. [24]. Femoral notching was considered if a 1-mm or deeper groove was cut into the femoral neck. The zones described by Gruen et al. [25], modified by Johnston et al. [26], were used to assess the femoral cementless components. The zones around the resurfacing femoral component were classified according to the criteria of Steffen et al. [2]. Acetabular fixation was described using the zones classified by DeLee and Charnley [27], stress shielding as per Engh et al. [24], and heterotopic ossification as per Brooker classification [28]. Fluoroscopic views were not used, but a standardized protocol with experienced radiology technicians was used.

Blood metal ions concentrations (cobalt and chromium) were measured according to accepted guidelines at 6 months, 2 years, and 10 years follow-up using ICP-MS technique [29]. Ion level thresholds were used according to the recommendations of the Medicines and Healthcare Products Regulatory Authority (MHRA) guidance released in 2012 and updated in 2017 (≥7 part per billion [ppb]; 119 nmol/L Co or 134.5 nmol/L Cr) [30].

In case of unjustified pain, a magnetic resonance imaging (MRI) scan was performed to evaluate for potential pseudotumor, defined as a solid/semi-solid periprosthetic soft tissue mass eccentric to the joint with a minimum diameter or 2 cm not otherwise attributable to infection, malignancy, or scar tissue [31].

Continuous variables were described using means and ranges. Categorical variables were described using absolute frequencies. To analyze differences between the HR group and the modular THA group in functional scores, implant survivorship, and metal ion levels, a two-tailed paired *t*-test and Wilcoxon signed-rank test were used. All statistical analyses were performed using jamovi v2.3.3.0 (Sydney, NSW, Australia). *p* values < 0.05 were considered significant.

## 3. Results

There were no significant differences in gender, BMI, and ASA score between the two groups (Table 1). Compared to the HR group, the modular THA group was significantly older in age (57 years vs. 46 years; *p* < 0.05). Mean follow-up was 11.8 years in the HR group (range, 10.1–14.1 years) and 11.4 years in the modular THA group (range, 10–13.3 years; *p* > 0.05.). In the HR group, mean implant head size was 51 mm (range, 42–58 mm) and the mean acetabular component size was 57 mm (range, 48–64 mm). In the modular THA group, the mean head size was 49 mm (range, 46–54 mm) and the mean acetabular component size was 56 mm (range, 54–60 mm).

In the HR group the overall survivorship from revision from any cause was 91.7% (22 of 24 hips). Two cases were revised within six months postoperatively due to periprosthetic fracture (neck fracture) after accidental fall and converted to a modular LDH MoM THA implant, therefore the survivorship from revision from periprosthetic fracture was 91.7% (22 of 24 hips). No other HR implants required subsequent revision at mean 11.8 years follow-up. Therefore, survivorship from implant failure and/or aseptic loosening and/or metal debris related problems was 100% (22 of 22 hips).

The overall survivorship from revision for any cause in the modular THA group was 86.7% (13 of 15 hips). One case (6.6%), 57 years old at time of index surgery, with a 56-mm acetabular component, 48-mm CoCr head, and Coral H010 femoral component, underwent revision for femoral component aseptic loosening and ARMD six years after surgery, and he was revised to a S-ROM^®^ Modular Hip System (DePuy Synthes, Warsaw, IN, USA) with a ceramic on polyethylene bearing. Therefore, the survivorship from implant aseptic loosening was 93.4% (1 of 15 hips). One other modular THA was revised for traumatic periprosthetic fracture. Therefore, the overall survivorship from revision for periprosthetic fracture was 93.4% (1 of 15 hips).

Regarding the radiographic analysis, among the 22 HR, femoral neck notching was reported in three cases (of 22 hips, 13.6%), RLLs were reported in one case in zone 1 (of 22 hips, 4.5%), and heterotopic ossifications (HO) were noted in two cases (of 22 hips, 9.1%), one grade 1 and one grade 2. Femoral neck thinning ≥2 mm was reported in 17 cases (of 22 hips, 77.3%) with a mean value of 4.4 ± 3.2 mm (range, 2–12.6 mm). Cup inclination was measured on antero-posterior pelvis x-ray with a mean value of 38.7 ± 5.6° (range, 30.3°–53°). In the modular THA group, radiolucent lines were reported in three cases (of 13 hips, 23.1%) in femoral zones 1 and 7. One of these, in zone 7, was progressive in a symptomatic hip with positive MRI scan for ALVAL lesion, therefore considered as clinically loose stem (then revised). In the other two cases, the RLL were considered stable and asymptomatic, and therefore carefully yearly evaluated.

Mean Oxford Hip Score was 42.5 ± 9.3 points (range, 18–48 points) and 43 ± 6.5 (range, 27 to 48 points) at final follow-up in the HR and the modular THA groups, respectively (*p* = 0.877). The mean WOMAC score was 9.2 (range, 0–54 points) and 8 points (range, 0–40 points) at final follow-up in the HR and in the modular THA groups, respectively (*p* = 0.806). The mean UCLA score was 6.3 (range, 3–10 points) and 6 points (range, 3–8 points) at final follow-up in the HR and in the modular THA groups, respectively (*p* = 0.865) (Table 3).

**Figure 1:**
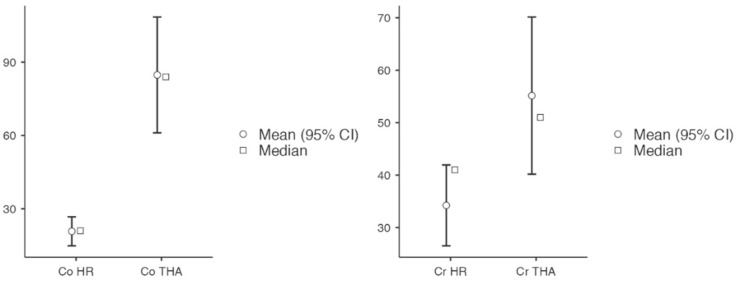
Cobalt and Chromium Ion Levels.

**Figure 2:**
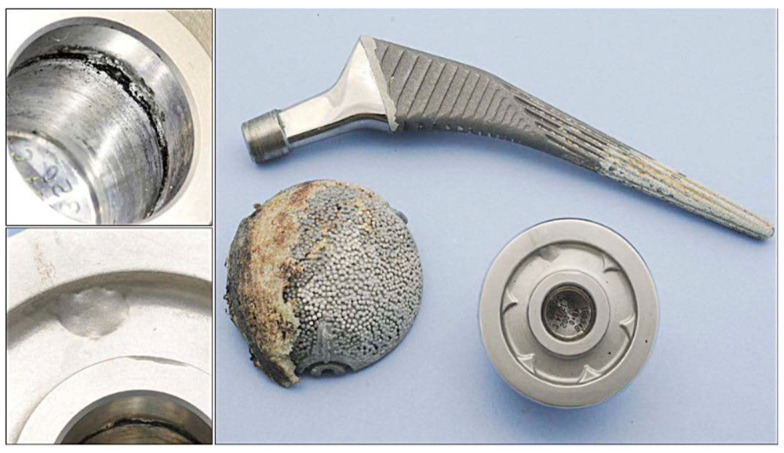
Retrieved analysis of revised modular MoM THA (56 mm acetabular component, 50 mm femoral head). (Picture from original publication on same patient series, Plant et al [20]).

Mean Cobalt levels in the HR group at final follow-up were 25.8 nmol/L (range, 10–55 nmol/L) compared to 89 nmol/L in the modular THA group (range, 29–140 nmol/L; *p* < 0.001) (Table 4, Figure 1). None of the patients in the HR group had cobalt levels above the threshold used during the articular surface replacement (“ASR”, Depuy, Warsaw, Ind) recall (119 nmol/L) while three patients (five hips) were above such a level in the modular THA group. Among them, two patients had bilateral modular MoM THA, and one patient with unilateral implant and increasing groin pain underwent MRI scan that reported ALVAL lesion, the implant was loose and revised. Mean chromium levels in the HR group at final follow-up were 33.5 nmol/L (range, 8–56 nmol/L) compared to 55.2 nmol/L in the modular THA group (range, 23 to 111 nmol/L; *p* = 0.026). None of the patients reported levels above the threshold of 134.5 nmol/L. Co/Cr mean ratio was reported to be 0.75 ± 0.46 in the HR group (range, 0.3 to 1.92), and in three cases (of 22 hips, 13.6%) reported to be above 1. On the other hand, the mean Co/Cr ratio was 1.73 ± 0.82 in the modular THA group (range, 0.41–2.80), being in seven cases (of 13, 53.8%) above 2. Of these, the highest level recorded was 2.80 and it was noted in the patient that underwent revision due to loosening and ALVAL lesion with crevice corrosion of the neck/head taper noted on retrieval analysis (Figure 2).

## 4. Discussion

This study reported the 10-year follow-up of a modular LDH MoM THA (withdrawn) and a well-established HR implant with similar functional outcomes and survivorship at final follow-up (>10 years).

The main reasons for revision were neck fracture in the HR group (8.3%, two of 24 hips) and periprosthetic fracture (6.6%, one of 15 hips) and stem aseptic loosening with ALVAL (6.6%, one of 15 hips) in the modular THA group. It is worth pointing out that, despite the limited number of patients, the Adept modular THA performed much better than other LDH MoM implant designs. The Durom acetabular component (Zimmer GmbH, Winterthur, Switzerland) was reported to have a failure rate for cup failure up to 11% at one year [32] and 15% at two years follow-up [33,34]. Similar results were described on the ASR XL THA (Depuy, Warsaw, Ind) with a cup failure rate up to 17% at three years follow-up [35,36]. Our results compare favorably considering a cup failure rate of 0% and a revision rate at 10 years due to aseptic loosening of 6.6% with a loose femoral component and well-fixed acetabular component.

The Adept HR is a well-established implant and considered a valid option in the case of selected young, active males. According to the last Australian Orthopaedic Association National Joint Replacement Registry report (AOANJRR), primary HR represented 1.2% of all hip replacement performed despite being 69.5% less than in 2005 [9]. In the last four years, the Adept HR has been the most used HR implant, in more than twice the number of cases compared to the following most used implant, the Birmingham Hip Resurfacing (BHR, Smith & Nephew Orthopaedics, Memphis, TN, USA) with an average of 282 and 139 implants per year, respectively.

The overall revision rate in the HR group was 8.3% (two of 24 hips), the revision rate for implant failure, AL loosening, and ARMD was 0% (0 of 24 hips), while the revision rate for periprosthetic fracture was 8.3% (two hips). Our results are in line with the AOANJRR that reported a revision rate at 10-years of 6.2% (range, 5.7–6.6%), while compare favorably in terms of main causes of revision as they are reported to be loosening (25.9%), metal related pathology (21.9%), and fracture (19.9%) [9]. These findings suggest the reliability of the Adept HR in terms of survivorship from complication related to implant fixation and adverse reactions to metal ions. Moreover, our results are in line with those reported by Stoney et al. [37], in a registry-based study that compared the revision rates of 4790 BHR with 2696 of the three best performing primary THA implants in men younger than 65 years. The authors reported higher revision rate for fracture during the entire analyzed follow-up time of 17 years in the BHR group (HR 2.57 (95% CI 1.24–5.33); *p* = 0.01).

The overall revision rate in the modular LDH MoM THA was 13.3% (two of 15 hips). However, the revision rate for aseptic loosening of the femoral component with associated ALVAL lesion was 6.6% (one hip) and the revision rate for periprosthetic fracture was 6.6% (one hip). These results, as previously mentioned, compare favorably with the literature on similar withdrawn LDH MoM THA implants both in terms of overall survivorship, survivorship from failure of the acetabular component and from ARMD [32,33,34,35,36,38], suggesting higher reliability and better kinematics with lower wear rate. No hip dislocations were reported overall, supporting the effects of LDH THA and HR in providing high stability of the hip joint, maximizing the ROM, as frequently reported in literature [39,40,41,42,43].

Serum metal ions levels are important to identify potential failure of the bearing and/or modular junction before catastrophic complications and severe soft tissue damages. Metal ion levels were reported to be higher in the modular THA group (Co 89 nmol/L vs. 25.8 nmol/L; Cr 55.2 nom/L vs. 33.5 nmol/L; *p* = 0.026) (Table 5). However, the small sample size and the number of bilateral implants in the modular THA group may have affected such a result. Moreover, none of the patient in the HR group had ion levels above the cutoff defined during recall while five hips (three patients) were above such levels in the modular THA group. Again, two of these patients had modular THA bilaterally and their levels have steadily increased since the midterm follow-up despite remaining asymptomatic. The Co/Cr ratio was reported to be higher in the modular THA group compared with the HR group (mean, 1.73 ± 0.82 vs. 0.75 ± 0.46), in line with results reported by Ridon et al. [44] when they compared 83 LDH THA with 90 HR at 10 years follow-up, reporting significantly higher Co and Cr serum levels in the former group (Cr, 1.75 µg/L vs. 1.07 µg/L; Co, 5.75 µg/L vs. 0.89 µg/L). Similarly, Hoti et al. [45], suggested that greater corrosion is correlated with higher Co/Cr ratios. These findings are in line with results reported in the recent literature (Table 6) [46,47,48,49,50], and reasonably explained by the additional articulation junction represented by the trunnion of the femoral neck and the femoral head taper, potentially leading to the additional release of metal ions due to mechanical wear, corrosion, or a combination of both, generally called trunnionosis [15]. This is supported by the retrieved findings of the revised Corail stem from the modular THA group (Figure 2). In addition, Co ions are more soluble and readily excreted, while Cr wear particles can precipitate in the form of “black surface deposit” in the surrounding tissues [44]. Therefore, the increased Co/Cr ratio for the modular THA is probably correlated with the corrosion of the modular junctions, in agreement with what previously reported [51,52,53]. It has been reported that higher frictional torques due to increasing femoral head size can lead to a greater corrosion at the taper junction [54,55,56,57].

Regarding the clinical outcomes, both groups reported excellent results at more than 10 years follow up using OHS (HR 42.5 ± 9.3, THA 43 ± 6.5; *p* = 0.877) and WOMAC score (HR 9.2, THA 8; *p* = 0.865). Despite being not significant, such results are in line with those reported by Kostretzis et al. [58] and Konan et al. [16], showing similar outcomes in modular MoM THA and HR at nine and 15 years follow-up.

In terms of radiographic outcomes, all components included for final analysis were considered to be stable. No cases of ARMD or ALVAL were detected in the HR group, comparing favorably with results reported by Hastie et al. [59], with a radiological incidence of ARMD of 34% at 13 years (out of 98 BHR). Overall, the total reported ARMD rate, including all MoM hips, was 2.6% (one of 39 hips), being 6.6% in the modular THA group (one of 15 hips) and 0% in the HR group (0 of 24 hips), comparing favorably with similar MoM implants discontinued together with the Adept MoM THA [32,33,34,35,36].

There are several limitations to this study. First, as a retrospective analysis, its nature makes it susceptible to selection and detection bias since patients were not randomized, postoperative evaluation was not blinded, and older and heavier patients might have been considered more suitable for a modular THA implant rather than a HR. Second, the small number of patients included in the study makes difficult to draw a definitive and clear conclusion concerning these implants, suggesting the interpretation of these results with all due caution. Third, the fact that the surgical procedures were performed by two different surgeons, despite their experience, may lead to a potential performance bias.

According to our results, the HR Adept implant reported excellent results at 10 years follow-up, in line with the registry data. The LDH modular THA with a Corail stem performed extremely better than the comparable withdrawn implants. Functional outcomes were comparable between the two groups, showing excellent performances. Survivorship of the implants was in line with that reported in the literature on similar designs, while the failure rate due to the MoM bearing and ARMD were considerably lower, in particular when referring to the other withdrawn modular THA implants. Therefore, we suggest the reliability of a well-known and established HR implant in selected young and active male patients, and report that the Adept modular MoM THA, despite being discontinued, is associated with excellent clinical outcomes and survivorship from failure for implant-related causes.

## Figures and Tables

**Table 1:** Baseline Patients Characteristics.

	Hip Resurfacing	SD (±)	(Range)	Modular Total Hip Arthroplasty	SD (±)	(Range)	*p*
Age [yrs]	46.3	6.7	30–63	56.6	6.4	44–62	<0.05
Weight [Kg]	89.5	17.2	58–134	98.7	14.1	73–119	0.065
Height [m]	1.76	0.1	1.58–1.95	1.78	0.1	1.68–1.92	0.261
BMI [Kg/m^2^]	28.4	4.5	18–39	30.1	2.5	26–33	0.100
ASA	1.6	0.6	1–3	1.4	0.5	1–2	0.657
Follow-up [months]	141	14.1	120–170	137	15.6	120–159	0.419

SD: Standard Deviation; BMI: Body Mass Index, ASA: American Society of Anaesthesiology Score.

**Table 2:** Indication of Index Surgery.

Indication of Index Surgery	HR (24 Hips)	THA (15 Hips)
Primary Hip OA	19 (79.2%)	10 (66.7%)
DDH	5 (20.8%)	-
Periprosthetic fracture	-	2 (13.3%)
Secondary OA	-	1 (6.7%)
AVN	-	1 (6.7%)
OA in Haemochromatosis	-	1 (6.7)

OA: Osteoarthritis; DDH: Developmental Dysplasia of the Hip; AVN: Avascular Necrosis; HR: Hip Resurfacing; THA: Total Hip Arthroplasty.

**Table 3:** Clinical Outcomes of Hip Resurfacing and Modular Total Hip Arthroplasty.

Implant (N)	Oxford Hips Score		WOMAC		UCLA	
	Mid-Term	Last	Mid-Term	Last	Mid-Term	Last
HR (22)	45 ± 3.9 (34–48)	43 ± 9.3 (18–48)	6 ± 9.4 (0–36)	9 ± 16.8 (0–54)	7 ± 1.9 (3–10)	6 ± 1.8 (3–10)
Modular THA (13)	41 ± 6.4 (27–48)	43 ± 6.5 (27–48)	13 ± 15.1 (0–46)	8 ± 12.5 (0–40)	6 ± 1.8 (3–9)	6 ± 2.1 (3–8)
*p*		0.877		0.806		0.866

HR: Hip Resurfacing; WOMAC: Western Ontario and McMaster Universities; UCLA: University of California and Los Angeles; THA: total Hip Arthroplasty.

**Table 4:** Ion Levels Details.

	Cobalt HR (nmol/L)	Chromium HR (nmol/L)	Cobalt Modular THA (nmol/L)	Chromium Modular THA (nmol/L)
N	20	20	13	13
Mean	22.3	33.5	84.8	55.2
Median	21.0	37.5	84	51
SD	13.8	14.9	43.6	27.6
Minimum	8	8	29	23
Maximum	55	56	140	111

HR: Hip Resurfacing, N: Number, SD: Standard Deviation, THA: Total Hip Arthroplasty.

**Table 5:** Comparison of Metal Ions Levels.

HR	Modular THA	Test	Statistic	df	*p*	Mean Difference	SE Difference
Co (nmol/L)	Co (nmol/L)	Student’s t	−5.78	12.0	<0.001	−64.0	11.07
		Wilcoxon W	0.0	<0.001		−65.0	11.07
Cr (nmol/L)	Cr (nmol/L)	Student’s t	−2.15	12.0	0.026	−20.9	9.75
		Wilcoxon W	17.0	0.024		−17.5	9.75

HR: Hip Resurfacing; THA: Total Hip Arthroplasty; Co: Cobalt; Cr: Chromium; SE: Standard Error; df: Degrees of Freedom.

**Table 6:** Ion Levels and Revision Rates at Mid-to-Longterm Follow-up.

Author (Year)	Type of Study	Hip Arthroplasty	Implant	Follow-Up (yrs)	Revision Rate (N)	Metal Ion Levels ± SD (Range)
Gani et al. (2022) [7]	Retrospective	105 HR	36 BHR 69 ADEPT	14.9	13.3% (14/105)	Co 26.6 nmol/L ± 24.5 Cr 30.6 nmol/L ± 15.3
Kearns et al. (2022) [46]	Retrospective	71 HR	BHR	12.7 ± 1.4	N/A	Co 3.12 ± 6.31 μg/L = 52.9 nmol/L Cr 2.62 ± 2.69 μg/L = 50.5 nmol/L
Pietiläinen et al. (2022) [47]	Retrospective	171 HR	BHR	7.5 (3.9–14)	N/A	Co 1.6 ppb (0.1–100) = 27.2 nmol/L Cr 1.5 ppb (0.2–63) = 28.9 nmol/L
Kostretzis et al. (2021) [58]	RCT	24 HR 24 LDH THA	Durom	15	HR: 8.3% (2/24) THA: 20.8% (5/24)	HR Co 1.7 μg/L ± 2 = 28.9 nmol/L HR Cr 1.4 μg/L ± 1.1 = 26.9 nmol/L LDH Co 3.8 μg/L ± 3.2 = 64.5 nmol/L LDH Cr 1.9 μg/L ± 1 = 36.6 nmol/L
Su et al. (2021) [48]	Retrospective	280 HR	BHR	10	7.1%	Co 1.3 ppb = 22.1 nmol/L Cr 1.4 ppb = 26.9 nmol/L
Høl et al. (2021) [49]	Retrospective	44 HR	BHR	5	N/A	Co 1.1 µg/L (0.4–6.3) = 18.7 nmol/L Cr 1.4 µg/L (0.4–11.7) = 26.9 nmol/L
Ridon et al. (2019) [44]	Retrospective	90 HR 83 LDH THA	Durom	10	THA 29.9% HR 2.3%	THA Co 5.75 μg/L (3.82–19.2) = 97.6 nmol/L THA Cr 1.75 μg/L (1.34–2.94) = 33.7 nmol/L HR Co 0.89 μg/L (0.67–2.89) = 15.1 nmol/L HR Cr 1.07 μg/L (0.67–1.65) = 20.6 nmol/L
Kiran et al. (2019) [50]	Retrospective	72 HR	ReCap	10	2.8% (2)	Co 28.83 ± 8.42 nmol/L Cr 39.93 ± 9.64 nmol/L

HR: Hip Resurfacing; THA: Total Hip Arthroplasty; LDH: Large Diameter Head; BHR: Birmingham Hip Resurfacing; N/A: Not Available; Co: Cobalt; Cr: Chromium; SD: Standard Deviation; yrs: years; N: Number; ppb: parts per billion; RCT: Randomized Controlled Trial.

## Data Availability

The data presented in this study are available on request from the corresponding author.
